# Imbalance between Th17 and Regulatory T-Cells in Sarcoidosis

**DOI:** 10.3390/ijms141121463

**Published:** 2013-10-30

**Authors:** Hui Huang, Zhiwei Lu, Chunguo Jiang, Jia Liu, Yanxun Wang, Zuojun Xu

**Affiliations:** 1 Department of Respiratory Medicine, Peking Union Medical College Hospital, Chinese Academy of Medical Sciences & Peking Union Medical College, Beijing 100730, China; E-Mails: pumchhh@126.com (H.H.); jiang.cg@hotmail.com (C.J.); liujiafree@163.com (J.L.); wangyanxun2008@163.com (Y.W.); 2 Department of Respiratory Medicine, Yijishan Hospital of Wannan Medical College, Wuhu 100730, China; E-Mail: sduzhiwei@yahoo.com.cn

**Keywords:** granulomatous disease, sarcoidosis, regulatory T cells, Th17 cells, corticosteroids

## Abstract

Sarcoidosis is a systemic granulomatous disease, which is thought to result from an aberrant immune response. CD4^+^ T lymphocytes play an important role in the development of granulomas. Previously, the immunopathogenesis of sarcoidosis was focused on Th1/Th2 disturbances. The aim of this study was to evaluate the balance between newer CD4^+^ T lymphocytes, *i.e*., Treg and Th17 cells. In our studies, a decrease in Treg cells and an increase in Th17 cells were observed in the peripheral blood and BALF of sarcoidosis patients. A significant increase in the Th17/Treg cell ratio was observed in sarcoidosis patients. After treatment with prednisone, the expression of Foxp3 mRNA was elevated in the peripheral blood, and expression of (ROR)γt mRNA showed a downward trend. These findings suggest that sarcoidosis is associated with an imbalance between Th17 and Treg cells in peripheral blood and BALF. Therefore, targeting the cytokines that affect the Th17/Treg ratio could provide a new promising therapy for pulmonary sarcoidosis.

## 1. Introduction

Sarcoidosis is a multisystem granulomatous disorder, with thoracic involvement occurring in more than 90% of sarcoidosis patients [1]. Accumulating evidence has suggested that CD4^+^ T cells, particularly CD4^+^ T helper 1 (Th1) lymphocytes, play a major role in immune-mediated development and accumulation of granulomas [2,3]. In addition to the traditional Th1 and Th2 subset populations of CD4^+^ T cells, recent reports have identified CD4^+^ CD25^high^ Foxp3^+^ regulatory T cells (Tregs) and Th17 cells as two new distinct subsets [4,5]. Previously, the immunopathogenesis of immune-mediated diseases, including sarcoidosis, was focused on Th1/Th2 disturbances [6,7]. Recent studies have shown that the aberrant relationship of Treg and Th17 cells is also involved in immune mediated diseases, including autoimmune arthritis [8], psoriasis [9], inflammatory bowel disease [10], systemic lupus erythematosus [11], and several others. However, it is not clear whether the Th17/Treg balance is affected in CD4^+^ T-cell mediated sarcoidosis.

Little is known regarding the potential role of Th17 and Treg cells and it remains controversial whether the circulating Treg population is elevated and functionally defective in sarcoidosis [12–17]. Therefore, the objectives of this study were to evaluate a potential imbalance of Treg and Th17 cells in serum and bronchoalveolar lavage fluid (BALF) of patients with sarcoidosis. Further, we examined any potential effects of glucocorticoid treatment on the Treg/Th17 ratio.

## 2. Results

### 2.1. Characteristics of Enrolled Sarcoidosis Patients

Presenting symptoms included fatigue (80%), weight loss (70%, ranging from 3 to 12 kg), dyspnea (60%), cough (50%), chest pain (30%), low-grade fever (10%) and rash (10%). One of the patients had hepatic sarcoidosis after liver biopsy. Six patients with sarcoidosis had impaired diffusing capacity for carbon monoxide (DLCO) ranging from 50% to 70% of predicted. There were three cases with impaired forced vital capacity (FVC), ranging from 63% to 72% of predicted. And three patients had a forced expiratory volume in 1 second (FEV1) to FVC ratio (FEV1/FVC) of less than 0.7.

### 2.2. Increased Th17 Population and Decreased Treg Population in Patients with Sarcoidosis

**Figure 1:**
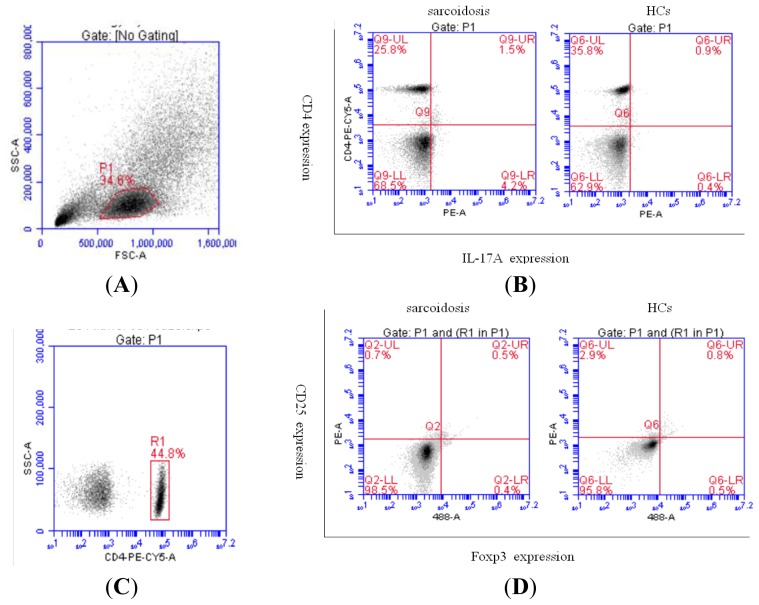
Representative plots of Th17 and Treg cells in sarcoidosis patients’ and HCs’ peripheral blood by flow cytometry (**A**, **B**, **C**, **D**). (**A**) Physical parameter (forward scatter and side scatter) of lymphocytes in the peripheral blood; (**B**) Representative dot plots showing CD4^+^ T cells producing IL-17A in sarcoidosis patients’ and HCs’ peripheral blood; (**C**) Physical parameter (side scatter) and CD4 expression of lymphocytes in the peripheral blood; (**D**) Representative dot plots showing CD4^+^ T cells producing CD25 and Foxp3 in sarcoidosis patients’ and HCs’ peripheral blood.

**Figure 2:**
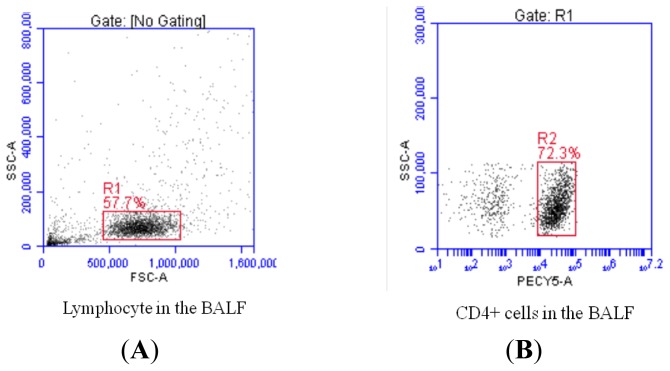

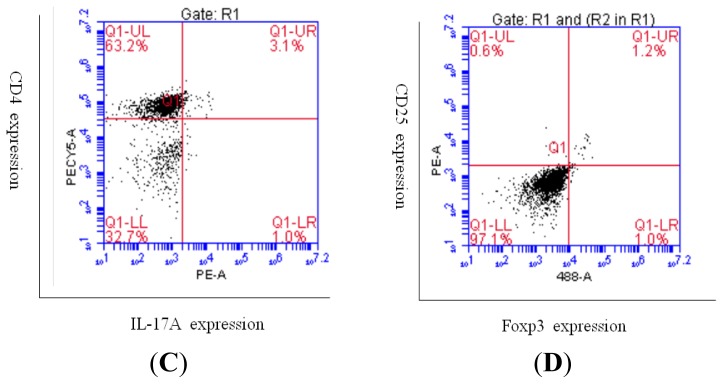
Representative plots of Th17 and Treg cells in sarcoidosis patients’ BALF by flow cytometry (**A**, **B**, **C**, **D**). (**A**) Physical parameter (forward scatter and side scatter) of lymphocytes in BALF; (**B**) Physical parameter (side scatter) and CD4 expression of lymphocytes in BALF; (**C**) Representative dot plots showing CD4^+^ T cells producing IL-17A in sarcoidosis patients’ BALF; (**D**) Representative dot plots showing CD4^+^ T cells producing CD25 and Foxp3 in sarcoidosis patients’ BALF.

We defined the phenotype of Treg cells as CD4^+^ CD25^high^ Foxp3^+^ cells and the phenotype of Th17 cells as CD4^+^IL-17^+^ cells. The peripheral blood mononuclear cells (PBMCs) from patients with sarcoidosis and healthy controls (HCs) (Figure 1) as well as BALF cells from sarcoidosis patients (Figure 2) were examined by flow cytometry for the Th17 and Treg subset populations, and the percentages of both cell populations were measured.

**Figure 3:**
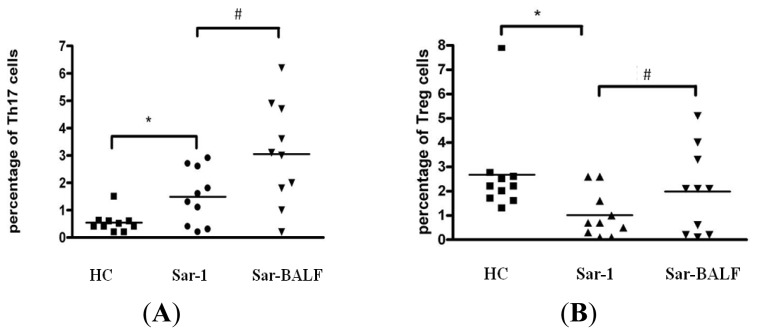
The percentage of Th17 cells (**A**) in the PBMC of HCs and sarcoidosis patients (Sar-1), and in the BALF of sarcoidosis patients (Sar-BALF) (*p = 0.0139; ^#^*p* = 0.0016). The percentage of Treg cells (**B**) in the PBMC of HCs and sarcoidosis patients (Sar-1), and in the BALF of sarcoidosis patients (Sar-BALF) (*p = 0.0286; ^#^*p* = 0.0346). Each datum point represents an individual patient sample. Median values for each group are represented by the horizontal line.

The Th17 cell population in PBMCs was increased in sarcoidosis patients (1.61% ± 1.09%) compared with HCs (0.51% ± 0.43%) (*p* < 0.05), whereas CD4^+^ CD25^high^ Foxp3^+^ Tregs were decreased in sarcoidosis patients (0.95% ± 0.91%) compared to HCs (2.68% ± 2.15%) (*p* < 0.05) (Figure 3A). In the sarcoidosis patients, the Th17 cell population in BALF (3.05% ± 1.87%) was increased compared with levels in PBMCs (1.61% ± 1.09%) (*p* < 0.05), while CD4^+^ CD25^high^ Foxp3^+^ Tregs in BALF were increased (2.19% ± 1.71%) compared to levels in PBMCs (0.95% ± 0.91%) (*p* < 0.05) (Figure 3B).

The mean fluorescence intensity (MFI) of Foxp3 was higher in Tregs of PBMCs from HCs (26169.1 ± 3258.3) compared to sarcoidosis patients (18347 ± 5958.8) (*p* < 0.05). There was no difference between the MFI of IL-17A in Th17 cells from HCs and sarcoidosis patients (2657.6 ± 1281.3 *vs.* 2300.9 ± 599.1, respectively) (*p* > 0.05).

### 2.3. An Imbalance of Th17/Tregs in Sarcoidosis

**Figure 4:**
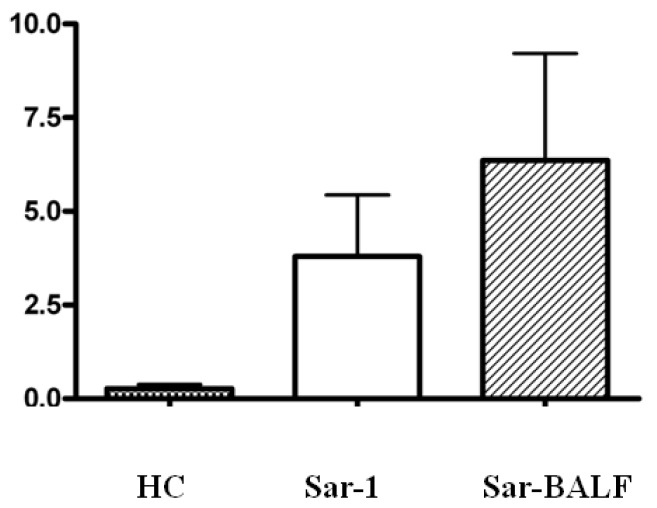
The ratio of Th17/Treg cells in healthy controls (HC) and sarcoidosis patients (Sar-1), and in the BALF of sarcoidosis patients (Sar-BALF).

Since we observed an increase in Th17 cells and a concomitant decrease in Tregs in sarcoidosis patients, we further explored this trend by investigating the balance of Th17 and Treg cells within the same patients and control subjects. The ratio of Th17 to Treg cells in the peripheral blood of HCs (0.27 ± 0.12) was less than the ratios in peripheral blood (3.80 ± 1.63) of sarcoidosis patients (*p* < 0.01); for the same individual sarcoidosis patient, however, there was no difference between the ratio of Th17 to Treg cells in peripheral blood and BALF (6.36 ± 2.86) (*p* > 0.05) (Figure 4).

### 2.4. Expression of Foxp3 and (ROR)γt in PBMCs from Sarcoidosis Patients

RORγt is an important transcription factor in the differentiation of Th17 cells, while Foxp3 functions as the master transcription factor in Treg cells. We investigated the expression levels of both transcription factors in PBMCs from sarcoidosis patients. The expression of Foxp3 was markedly increased after patients were treated with corticosteroids (0.0313 ± 0.002) compared with initial levels in the same patients (0.005 ± 0.0007) (*p* < 0.05). In contrast, in the same patients, the levels of RORγt expression trended towards a decrease after corticosteroids (0.16 ± 0.07) compared to initial levels (0.95 ± 0.36), but the difference was not statistically significant (*p* > 0.05).

## 3. Discussion

Sarcoidosis is characterized by the presence of noncaseating granulomas, but the pathogenesis of the disease remains poorly elucidated. It is now well established that T cells, and especially CD4^+^ helper cells, are mandatory in the development and maintenance of pulmonary granulomas in sarcoidosis. Traditionally, sarcoidosis has been associated with a highly polarised Th1 profile. This is demonstrated by the increased blood, BALF, and sputum Th1-associated cytokines, as well as chemokine and cytokine receptors. Also, there are low levels of Th2-associated cytokines as well as chemokine and cytokine receptors in blood, BALF, and sputum samples of patients with sarcoidosis [18–22].

Th17 lymphocytes are a distinct subset of CD4^+^ IL-17-producing T cells, and RORγt plays a central role as the master transcription factor in these cells [10,23]. Th17 cells play a critical function in the development of various autoimmune diseases and in mediating host defense mechanisms against a variety of infectious diseases [12,23]. Only a few reports to date have focused on the role of Th17 cells in sarcoidosis. Facco *et al.* found that Th17 cells infiltrate the lungs in sarcoidosis, localizing around and inside the granuloma at the sites of disease activity, not only in the early phase, but also in the progression towards the fibrotic phase of the disease [24]. In our study, we also found that the proportion of Th17 cells was elevated both in the peripheral blood and in the BALF of sarcoidosis patients.

Treg cells, defined by the expression of CD4, CD25, and the key transcription factor Foxp3 [25,26], have an important role in immune suppression and protection from autoimmune diseases [5,27]. Defects in the number and function of Treg cells, as well as a resistance of effector T cells to Treg cell-mediated suppression, could contribute to a failure of T cell regulation. Each of these defects has been shown to contribute to the development of autoimmunity in various model systems [28]. Analysis of the CD4^+^ CD25^+^ Treg population in sarcoidosis patients has produced conflicting data. In particular, the majority of studies reported an increased frequency and impaired function of CD4^+^ CD25^+^ Tregs in the active phase of sarcoidosis compared with healthy controls, and significantly increased levels of Treg cells in BALF [12–15,29]. These studies suggest that Treg cells in sarcoidosis patients are impaired in their ability to suppress the development of granulomas, and the resistance of pathogenic effector T cells to suppression by Treg cells is due to sarcoidosis. In our study cohort, the proportion of Treg cells in PBMCs was reduced in sarcoidosis patients compared with controls, which is similar to the results of Crouser *et al.* and Idali *et al.* [17]. Furthermore, as the MFI of Foxp3 was also lower in sarcoidosis patients, we hypothesize that Treg cell function in sarcoidosis patients may be impaired.

The balance between Th17 and Treg cells has been suggested as a new paradigm for autoimmune disease [30]. Notably, recent studies have identified this imbalance in several diseases, including systemic lupus erythematosus [11], rheumatoid arthritis [8], inflammatory bowel disease [10], primary biliary cirrhosis [31], idiopathic dilated cardiomyopathy [32], and others. Correction of the Treg/Th17 imbalance may have a therapeutic impact for patients with these diseases. Our study demonstrated an elevation of Th17 cells and a decrease of Treg cells in peripheral blood of sarcoidosis patients, leading us to conclude that there is a significant imbalance between Th17 and Treg cells in these patients. After treatment with corticosteroids, all ten patients showed a good response, and though the difference was not statistically significant, RORγt mRNA levels in the peripheral blood showed an obvious downward trend. At the same time, the level of Foxp3 mRNA was significantly elevated. These findings suggest that the imbalance between Th17 and Treg cells may contribute to the development of sarcoidosis. The alteration of the index of Th17/Treg cells likely indicates the therapeutic response in the clinic. Moreover, strategies designed to alter the cytokine milieu to favor Treg expansion and suppression of Th17 differentiation may be a novel and effective approach for the treatment of sarcoidosis.

## 4. Experimental Section

### 4.1. Patients

Ten Chinese sarcoidosis patients (9 females, 1 male; mean age ± standard deviation (SD): 51.9 ± 7.6 years) were enrolled from the Department of Respiratory Disease at Peking Union Medical College Hospital. All ten of the patients were non-smokers and had no prior smoking history. Diagnosis of sarcoidosis was established according to the typical chest radiographic imaging manifestations, compatible clinical features, and noncaseating granulomas on biopsy, with all other causes of granulomas ruled out. According to Scadding staging system, all of them were classified as stage 2.

At the time of sample collection, none of the patients had received treatment with corticosteroids or other immunosuppressants in the three or more months preceding the study. Bronchoscopy and bronchial alveolar lavage (BAL) was performed for all ten patients. Patients were prepared for the bronchoscopy with aerosolized lidocaine sprayed into the nasal cavity and oropharynx. During the bronchoscopy and bronchoalveolar lavage, patients were kept awake without any sedatives and vital signs were monitored throughout the procedure. A total of four 25 mL aliquots of normal saline solution were instilled separately and were immediately aspirated into a trap. The first aspirate was discarded and the second, third, and fourth aspirate were pooled. The total volume of BALF was recorded and was centrifuged.

All patients needed to be treated with corticosteroids according to the suggestions by the British Thoracic Society (BTS) in 2008 [33]. All patients were diagnosed with sarcoidosis for the first time. After treatment with prednisone (0.5–0.8 mg/kg/day) for four to six weeks, all patients had a good response: enlarged lymph nodes were reduced to one-third to one-half of their initial size, and more than half of the lung shadows disappeared.

Subjects without chest involvement were excluded. Ten sex- and age-matched healthy Chinese volunteers (9 females, 1 male, mean age ± SD: 51.9 ± 7.6 years) were recruited as HCs group. None of the volunteers in the control group had any prior disease, such as hypertension, diabetes mellitus, or chronic obstructive pulmonary disease (COPD). The study was approved by the ethics committee of Peking Union Medical College Hospital. Patients and volunteers provided informed consent for participation in the study.

### 4.2. Cell and Sample Preparations

Blood samples (20 mL) were obtained from all subjects in a fasting state and collected into test tubes using ethylenediaminetetraacetic acid (EDTA)-Na2 (BD-Plymouth, Devon, UK) as an anticoagulant. All sarcoidosis patients had their blood drawn twice: the day they were accepted for bronchoscopy and after being treated with corticosteroids for four to six weeks. Samples were divided into two parts: one for cell staining analysis and the other for RNA extraction. Plasma was stored at −80 °C. PBMCs were obtained by Ficoll density gradient (G&E Healthcare, Uppsala, Sweden). BALF and the cells in BALF were obtained by a speed centrifuge method. BALF samples were stored at −80 °C, and cells in BALF were prepared for staining.

For Th17 and Treg cell analysis, PBMCs were suspended at a density of 2 to 5 × 10^6^ cells/mL in complete culture medium (RPMI-1640 supplemented with 100 U/mL penicillin, 100 mg/mL streptomycin, 2 mM glutamine, and 10% heat-inactivated fetal calf serum; Gibco). The cell suspension was transferred to each well of a 24-well plate. Cultures were stimulated with phorbol myristate acetate (PMA, 30 ng/mL) plus ionomycin (750 ng/uL) for 5 h in the presence of 5 μg/mL brefeldin A (1.7 μg/mL; all from Sigma-Aldrich, St. Louis, MO, USA). The cells in BALF were prepared for staining using the same method. The cells were cultured at 37 °C in a 5% CO_2_ environment.

### 4.3. Flow Cytometric Analysis

PBMCs and BALF cells were processed for staining by the same method. For Th17 analysis, cells were first surface-stained with phycoerythrin-Cy5 (PECy5)-conjugated anti-human CD4 antibodies for 20 min, fixed and permeabilized with Perm/Fix solution, and then stained with phycoerythrin (PE)-conjugated anti-human IL-17A. For Treg analysis, the cells were incubated with PECy5-conjugated anti-human CD4 antibodies and PE-conjugated anti-human CD25 for 20 min at 4 °C in the dark. After surface staining, the cells were permeabilized and fixed, and stained with 488 anti-human Foxp3 according to the manufacturer’s instructions. Isotype controls were used to correct compensation and confirm antibody specificity. All antibodies were purchased from eBioscience (eBioscience, San Diego, CA, USA). Stained cells were analyzed by flow cytometric analysis using an Accurri C6 cytometer equipped with CFlow Sampler Analysis software (BD, Franklin lakes, NJ, USA).

### 4.4. Real-Time PCR Analysis

Total RNA was extracted with TRIzol (Invitrogen, Carlsbad, CA, USA) according to the manufacturer’s instructions. All samples were treated with DNaseI to eliminate potential genomic DNA contamination. The quality and quantity of the RNA were determined by ultraviolet spectrophotometer. The cDNA was synthesized using random hexamer primers and RNase H-reverse transcriptase (Invitrogen). RNA and cDNA were stored at −80 °C until further processing. TaqMan primers and probes for human (ROR)γt and Foxp3 were purchased from Roche (Real-time PCR Kits, Roche, Switzerland) and samples were analyzed using the Roche 480II Real-time PCR Detecting System (Roche, Switzerland). The following primer pairs were used for PCR analysis: (ROR)γt, F: 5′CCAAGGCAGGGCTCAATG3′ and R: 5′GAAGTCCACATCGGTCAGG3′; Foxp3, F: 5′GCCACAACCTGAGTCTGC3′ and R: 5′GTTCGTCCATCCTCCTTTCC3′; and 18S, F: 5′TGAGAAACGGCTACCACATC3′ and R: 5′TCCCAAGATCCAACTACGAG3′. PCR conditions for gene amplification began with a 10 min 95 °C enzyme activation step, followed by 40 cycles of 95 °C for 15 s and 60 °C for 30 s. The mRNA levels were normalized to the level of the 18S mRNA control. All samples were treated according to identical protocols and in parallel.

### 4.5. Statistical Analysis

Data was analyzed using the Statistical Analysis System (SAS) version 9.0 software package (SAS Institute Inc., Cary, NC, USA). Quantitative variables were described using mean ± SD and categorical data using frequency and percentage in the text and figures. Comparisons between two groups were analyzed by student’s *t*-test when data was normally distributed. A group *t*-test was used for group data, and a paired sample *t*-test was used for paired data. The Kruskal–Wallis test was used when data was not normally distributed. A value of *p* < 0.05 was considered to be statistically significant. Data was analyzed and graphed using Graphpad Prism v4 software (GraphPad Software, San Diego, CA, USA).

## 5. Conclusions

In summary, we found an increased Th17/Treg ratio in the peripheral blood and BALF in active sarcoidosis patients. After a good response to corticosteroid treatment, the imbalance of Th17/Treg cells in sarcoidosis patients shifted towards baseline. These results indicate a role for the imbalance of Th17 and Treg cells in the pathogenesis of sarcoidosis, and suggest that strategies designed to favor Treg cell expansion and suppression of Th17 cell differentiation may be valuable approaches for the treatment of sarcoidosis.
